# The protective role of melatonin under heavy metal-induced stress in Melissa Officinalis L.

**DOI:** 10.3906/kim-2012-7

**Published:** 2021-06-30

**Authors:** Elvisa HODŽIĆ, Semira GALIJAŠEVIĆ, Milica BALABAN, Sebila REKANOVIĆ, Halid MAKIĆ, Biljana KUKAVICA, Dijana MIHAJLOVIĆ

**Affiliations:** 1 University of Bihać, Biotechnical faculty, Luke Marjanovića Bosnia and Herzegovina; 2 Sarajevo School of Science and Technology, Sarajevo Bosnia and Herzegovina; 3 University of Banja Luka, Faculty of Natural Sciences and Mathematics, Banja Luka Bosnia and Herzegovina; 4 University of Banja Luka, Faculty of Agriculture, Banja Luka Bosnia and Herzegovina

**Keywords:** Melatonin, heavy metal, stress, lemon balm, antioxidant system

## Abstract

Heavy metals, due to their inability to degrade, pose a serious environmental and nutritional problem. The accumulation of essential and non-essential heavy metals in living organisms reduces normal growth and development, resulting in acute poisoning, disease and even death of organisms. Melatonin is a very important multifunctional molecule in protecting plants from oxidative stress due to its ability to directly neutralize reactive oxygen species (ROS). Also, melatonin has a chelating property, which may contribute in reducing metal-induced toxicity. In this paper, the protective role of melatonin in counteracting metal-induced free radical generation was highlighted. Using the HPLC-FLD technique melatonin was identified and quantified in the roots and leaves of lemon balm ( *Melissa officinalis* L.), grown under photoperiod conditions. Furthermore, the response of plants pre-treated with exogenous 0.1 mM melatonin to the increased zinc (Zn) and cadmium (Cd) concentrations was observed, with changes in mineral (Ca, Mg), physiological and antioxidant status of the plant during heavy metals stress. The obtained melatonin concentrations were the highest published for dry plants so far. Elevated Cd and Zn levels in soil caused alternation in biochemical and physiological parameters of lemon balm leaves and roots. However, melatonin pre-treatment increased plant tolerance to heavy metals stress. Increased Cd and Zn uptake and their translocation into the leaves were also improved, indicating the possible use of melatonin in phytoremediation.

## 1. Introduction

Melatonin ( *N* -acetyl-5-methoxytryptamine) is indolamine, widespread in the animal world [1]. In vertebrates it plays a very important role as a hormone, especially neurohormone. Since the discovery and detection in plants in 1995 [2,3], melatonin has attracted a great deal of research attention. In recent years, it has been identified and quantified in more than 140 plant species [4,5]. 

Because plants are sessile, they have to withstand many stresses throughout their life cycle. When exposed to a stressful environment, rapid and tremendous change must occur within plant cells in order to survive. 

Heavy metals like Zn and Cu are essential for normal plant growth, but their excess is toxic. A metal ion binds to a sulfhydryl group in enzymes and other proteins by inhibiting their activity or disrupting their structure [6]. Furthermore, heavy metals cause oxidative damage to biomolecules by triggering free radical-mediated chain reactions resulting in lipid peroxidation, protein and nucleic acid oxidation [7].

Melatonin is an amphiphilic molecule that very easily diffuses through cell membranes into the cytoplasm and enters subcellular compartments [1]. A number of studies have highlighted the importance of melatonin in regulating almost all stages of plant growth and development (from seed germination to leaf aging), as well as defense against various biotic and abiotic environmental stresses (drought, salinity, extremely low or high temperatures, various pathogens, and chemical agents) [1,8–10]. The effective role of melatonin in reducing stress in plants can most often be attributed to increased photosynthesis, enhancement of cellular redox homeostasis and alleviation of oxidative stress, and regulation of the expression of genes responsible for stress [11]. 

As abiotic stress factors, heavy metals disrupt fundamental metabolic processes leading to anatomical, morphological, and biochemical disorders. Metals also exert their toxic effects in such way that they lead to increased production of reactive oxygen species (ROS) that impair the redox homeostasis of the cell [12]. Phenolic compounds also exert a very important antioxidative function [13–16]. They have the potential to directly scavenge ROS and RNS (reactive nitrogen species), but also present endogenous substrates for vacuolar and apoplastic class III peroxidases. Also, phenolic compounds along with the peroxidases are involved in H_2_O_2_ scavenging, which accumulates in the apoplast and vacuoles [14,17,18].

The identification and quantification of melatonin, as well as the analysis of the biochemical characteristics of lemon balm under the influence of heavy metals and exogenous melatonin, have not been conducted so far [19]. This paper examines the role of melatonin as a signaling molecule in signaling from the environment and mediating the response of plants exposed to the maximum allowable concentrations of cadmium and zinc. The study evaluated the effect of exogenously added melatonin on its endogenous level in the *Melissa officinalis* L. leaves and roots, medicinal plant known for beneficial effects on insomnia and depression [20]. Biological responses to the presence of metals by melatonin accumulation or by alteration of the antioxidant enzymes activity in the lemon balm roots and leaves were also compared. In addition to its antioxidant role, melatonin can create complexes with metals and thus exert its protective role. 

## 2. Materials and methods

### 2.1. Plant cultivation, treatments, and growth conditions

**Figure 1:**
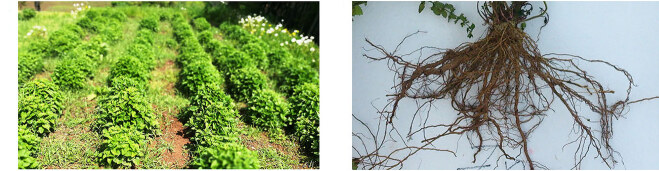
Lemon balm plants in the open field conditions (A) and plant root branching (B).

Lemon balm seeds were rinsed under running and distilled water, covered with air-dried conventional substrate and daily sprayed with distilled water for the control and 0.1 mM melatonin solution, until germination was complete (30 days), after which the seedlings were sown in an open field, and treated with 3 g/L zinc sulfate and 15 mg/L cadmium sulfate solution. The treatment was repeated three times. Open field conditions in the terms of photo-period were applied as shown in Figure 1.

The temperature in the growth period varied from 15.5 to 22.9 °C, according to the data collected from the State Hydro Meteorological Institute in Bosnia and Herzegovina. Lemon balm samples for the melatonin analysis were harvested in June (65 days old plants), before flowering, lyophilized at –50 °C for 25–30 h (VaCo 2, ZIRBUS Technology, GmbH, Germany) and stored at 4 °C till extraction. 

### 2.2. Melatonin analysis

Direct extraction procedure was chosen for melatonin extraction, which has proven to be the best with methanol as an extraction agent [21]. All sample preparation processes were performed under dark artificial light, due to the possible degradation of the analyte [3]. Lyophilized and grounded lemon balm roots were weighed in test tubes with methanol in a total volume of 10 mL. The samples were left 15–17 h at 4 °C, with shaking, after which a 30 min ultrasonic treatment at 4 °C (WiseClean WUC, Witeg GmbH, Germany) was performed (water and ice mixture). The tubes with plant tissues were centrifuged at 6000 rpm for 30 min (Alresa Mod, Digicen). The extract of each sample was evaporated to dryness under vacuum (Rotavapor R-215, Buchi Switzerland). The residue was redissolved in 1 mL of methanol, transferred to vials and stored at 4 °C. The samples were filtered (0.45 μm) and analyzed by high-pressure liquid chromatography (Agilent 1100 Series) coupled with a fluorescence detector (FLD), reversed phase C18 gravity column (Nucleodur, 3 μm particle diameter, 150 mm × 4 mm, Macherey-Nagel, Germany) and integrated pre-cell as well as programmed mobile phase 20% methanol: 80% water. The flow rate of the analyte was 1.5 mL/min, at room temperature.

### 2.3. Heavy metal and macroelements determination

Heavy metal contents were determined by using atomic absorption spectrophotometer (AAS), Perkin Elmer Analyst 400, after the acid digestion of the examined samples. 0.3 g of the lyophilized plant material (leaves and roots) were digested with 3 mL of concentrated HNO_3_, 3 mL of H_2_O_2_, and 1 mL of concentrated HCl in closed polytetrafluoroethylene (PTFE) vessels in a microwave oven. After cooling, the content was transferred to a 50 mL volumetric flask, completed with deionized water, filtrated through a Whatman No44 filter paper into pure flasks and analyzed by method of the flame atomic absorption spectrophotometry. All determined metals were atomized in an oxidizing light blue flame formed by mixture of compressed air (10 L/min) and acetylene (2.5 L/min). Contents of Cd, Zn, Mg, and Ca were established respectively at the wave lengths: 228.8, 213.7, 285.2, and 422.7 nm, using the deuterium background for correction of signal for Cd and Zn. All calibration standard solutions were prepared by dilution of the single-standard stock solution (1000 mg/kg, Perkin Elmer) with 10% HNO_3_. Quality control of the extraction and determination of elements was carried out by analysis of certified reference material (ERM^®^-CD218, rye grass) with recoveries of metal concentrations for Cd, Zn, Mg, and Ca in the following order: 94.2; 96.1; 90.3, and 103.9%. All extractions of metals from the analyzed samples, as well as the measurements of their contents were done in three replicates. All laboratory vessels used for determination of metal contents were prewashed in the 10% HNO_3_ and then washed with deionized water.

### 2.4. Total phenolic and flavonoid content determination

Total phenolic content was determined according to the Folin–Ciocalteu colorimetric method in alkaline medium [22]. The gallic acid was used for the standard curve, and the reduction of Folin–Ciocalteu reagent by the samples was expressed as mg of gallic acid equivalents (GAE) per g of extract. The flavonoid content was determined by the Chang et al. method [23], which is based on the flavonoid ability to form metal complexes in the presence of metal ions (e.g. AlCl_3_), extend delocalization and move UV and vis absorption bands by about 50 nm toward higher wavelengths (bathochromic effect).

### 2.5. DPPH scavenging activity

The antioxidant activity of the extracts was measured on the basis of the stable 1, 1-diphenyl 2-picrylhyorazyl (DPPH) free radical scavenging activity according to the method described by Soler-Rivas et al [24]. Briefly, 1 mL of 0.4 mM DPPH solution in 1 mL of methanol was mixed with 1 mL of plant extract solution of varying concentrations (50, 100, 150, 200, and 250 µg/mL). The reaction was carried out in triplicate and the decrease in absorbance was measured at 515 nm after 30 min in dark using PhotoLab 6600 UV-vis spectrophotometer (Xylem Analytics Germany Sales GmbH & Co. KG, WTW). 

### 2.6. Ferric reducing/antioxidant power (FRAP)

The Benzie and Strain method [25], was used to determine the ferric reducing antioxidant power. Substances with reduction potential react with potassium ferricyanide to form potassium ferrocyanide, which then reacts with ferric chloride to a form ferric-ferrous complex with an absorption maximum at 593 nm.

### 2.7. CUPRAC capacity

The cupric reducing antioxidant capacity of plant extracts was determined according to the method of Apak et al [26]. Reaction mixture of 1 mL CuCl_2 _(0.01 M), 1 mL of acetate buffer (1mM, pH = 7), 1 mL of ethanolic solution of neocuproin (7.5 × 10^–3 ^M), and 0.5 mL of ethanol extract was left for 30 min at room temperature. The absorbance solution was spectrophotometrically measured at 450 nm relative to the reference solution containing deionized water instead of sample. Trolox was used as a standard, and the reduction potential was expressed as TEAC_CUPRAC_ in mmol equivalents of trolox g dry matter (mmol/L).

### 2.8. Total soluble protein content measurement

Total soluble protein content was determined by Lowry et al. [27] with bovine serum albumin as standard. For protein and enzyme activity determination lyophilized leaves and roots were powdered in liquid nitrogen and kept at –20°C to analysis. For extraction 0.5 g of the sample and 4 mL of extraction buffer was mixed. Sodium phosphate buffer (0.1M, pH 6.4), 0.1 mM PMSF (phenylmethylsulfonyl fluoride), and 0.2% TWEEN (polyethylene glycol sorbitan monolaurate) were used. After homogenization, the samples were centrifuged for 10 min at 10,000 rpm and 8 °C. One mL of supernatant was separated with addition of 5% PVP (polyvinylpyrrolidone), and after vortexing, centrifugation was repeated for 10 min at 10,000 rpm. The supernatant was used to analyze soluble proteins, measuring the absorbance at 550 nm. 

### 2.9. Spectrophotometric determination of peroxidase activity (E.C. 1.11.1.7)

A modified Teisseire and Guy method [28], was used for the peroxidase activity assay. The increase in absorbance at 430 nm (ε430 = 12 mM^–1^cm^–1^) was monitored. The reaction was initiated by adding 3.43 mM H_2_O_2_ to a mixture containing 50 μL of sample, 10.3 mM pyrogallol and Na-phosphate buffer (pH 6.4) at 37 °C. The activity of peroxidases is expressed as μmol min^–1^mg^–1^ protein.

### 2.10. Statistical analysis

The data were analyzed using SPSS Statistics v: 23.0 Analysis of variance (ANOVA) was conducted and significance of differences among treatments and in time dependence were tested using the least significant difference (LSD). Differences were significant at the *p < 0.05 probability level.

## 3. Results and discussion 

To gain an insight into the effect of cadmium and zinc contaminated soil on the plant antioxidative activity, total phytohormone content the variability of melatonin and the difference in biological response to abiotic stress were determined. An effect of exogenous melatonin addition on lemon balm antioxidative activity was investigated by measuring total phenols, flavonoids, protein content, antioxidants, and activity of class III peroxidases. 

**Figure 2:**
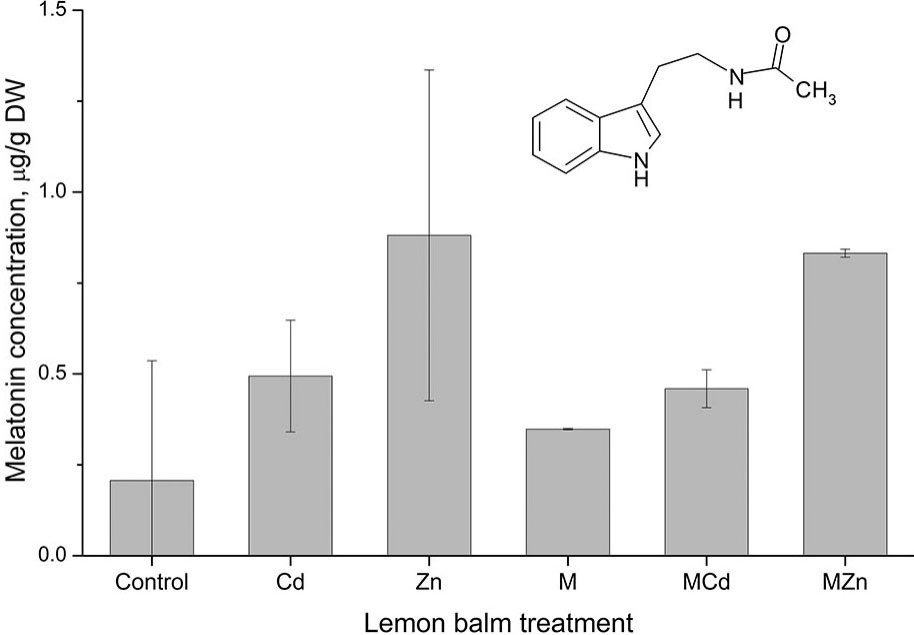
Melatonin content in lemon balm roots and leaves under Cd, Zn treatments and melatonin pre-treatment.

Figure 2 shows the effect of the different treatments on the endogenous melatonin level in the lemon balm roots. Treatments had the significant effect on the endogenous melatonin concentration (p < 0.5). There was an increase in the content of melatonin under the influence of higher concentrations of both Cd and Zn, and even more significant increase in samples pre-treated with melatonin during plant growth and development, but also in combination with increased concentrations of Cd and Zn compared to controls. 

As can be seen, ZnSO_4_ had the highest stimulatory effect on the melatonin content with and without melatonin pre-treatment, with 75% and 73% of endogenous melatonin increasement, followed by CdSO_4_. Similar results of melatonin concentration were obtained earlier in lemon balm leaves, with the same heavy metal treatment and three times higher endogenous melatonin concentration. The concentration is three times higher, which is expected since the leaves show greater antioxidant activity [29]. The authors have emphasized that melatonin might protect plants from stress conditions and to prevent injuries induced by oxidative stress at the cellular level, by elevating the activity of antioxidative enzymes in first growing phase of the plants [19].

Zinc, usually required for the synthesis of auxin (a growth-regulating component), seems to have influence on the melatonin synthesis. Likewise, zinc is essential in the synthesis of tryptophan as a precursor in the biosynthesis of indole acetic acid and melatonin [30]. Accordingly, an increase in the tryptophan content of zinc-fertilized rice grains in limestone soils has been observed [31]. Also, Tan et al. [31] demonstrated that melatonin added to soil enhanced the tolerance and survival of pea plants ( *Pisum sativum* L.) against copper contamination, indicating that the presence of melatonin in plants can be used in phytoremediation. There is ample evidence to confirm that heavy metal ions have a major influence on the content of compounds with hormonal function. Thus, Zn ions in the root of *Lupinus albus* L. cause an increase of endogenous amounts of melatonin (12 times higher than the control) [32].

**Figure 3:**
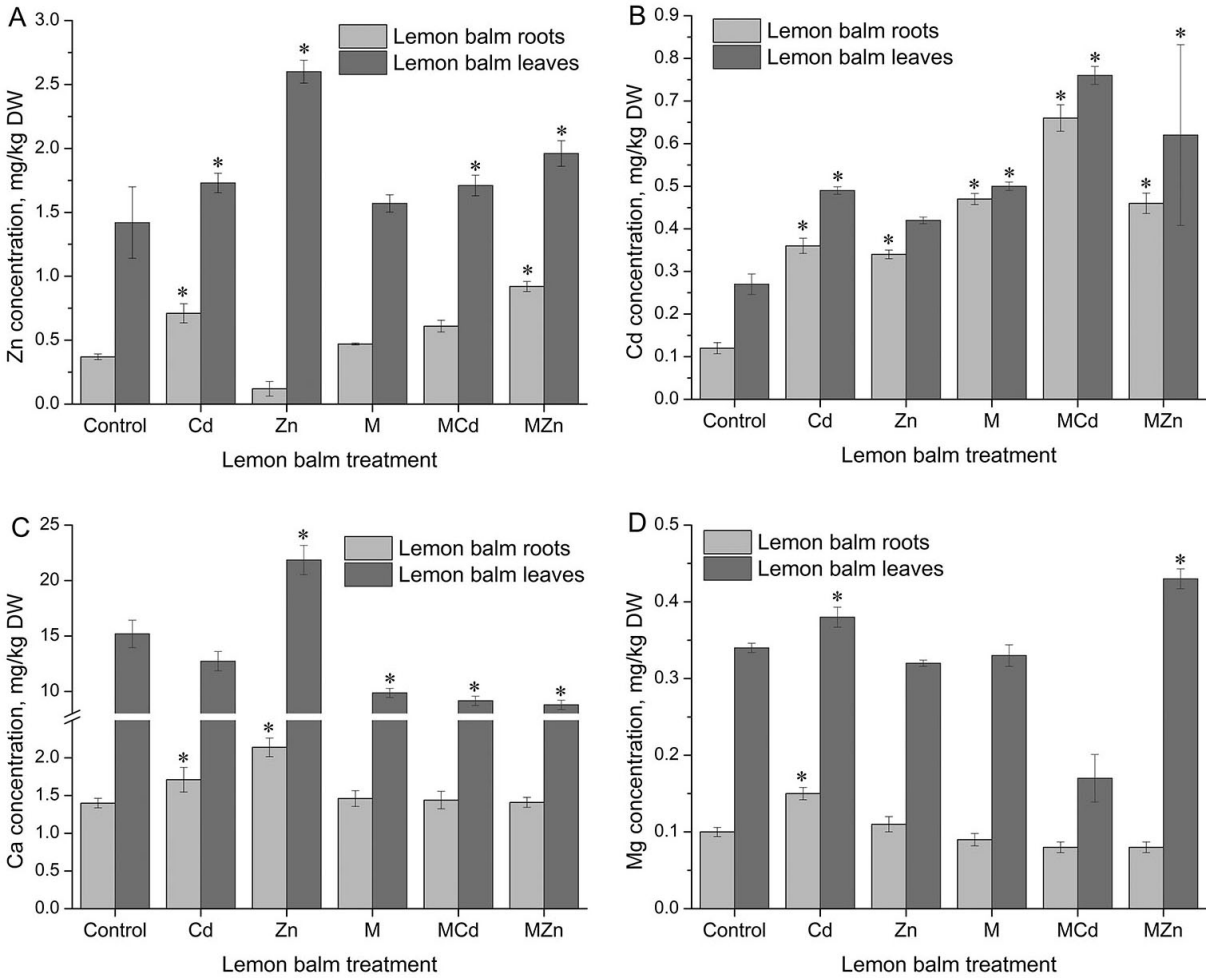
Concentration of Zn (A), Cd (B), Ca (C), and Mg (D) in lemon balm roots and leaves. The data are presented as means of three replicates. Dots marked with an asterisk differ significantly (Tukey test; p < 0.05). M - melatonin.

Plants have developed numerous mechanisms in the binding of metals from the soil and their transport within the plant itself. The introduction of metal into the root of a plant is a complex process that involves the transfer of metal from the soil to the root surface and within the root cells. Similar to copper, zinc is actively absorbed from the soil, with high levels of calcium and magnesium adversely affecting its uptake. After analyzing the content of heavy metals in lemon balm leaf and root, it is evident that higher concentrations of Zn were adopted by the leaf. From Figure 3A a linear trend of increasing Zn concentration in the leaves can be observed in following order: control <Cd <Zn. However, the most significant increase in the concentration of this metal relative to the control was recorded by the roots of plants pre-treated with exogenous melatonin and treated with Zn ions (0.922 and 2.1 mg/kg Zn). By accumulating 2 and 2.5 times higher concentrations of Zn in the leaves of tested plants pre-treated with melatonin, this could be an effective way of phytoextraction, i.e. phytoremediation of soil contaminated with this heavy metal.

Results showed that the soil contained 0.41 mg/kg of Cd even before the addition of the cadmium (Figure 3B). Higher concentrations of Cd^2+^ were observed in all three treatments with exogenously added melatonin (melatonin, melatonin + Zn^2+^, melatonin + Cd^2+^). Although calcium and zinc reduce Cd uptake, and its transport is highly dependent on the concentration of this element in the environment, the presence of zinc in our samples did not reduce cadmium uptake, except slightly in the roots of plant pre-treated with melatonin. This may be due to the relatively low zinc concentrations observed in both, lemon balm root and leaf. Zn-treated lemon balm plants show higher Cd accumulation than control: 0.344 mg/kg in roots and 0.416 mg/kg in leaves. Melatonin pre-treatment caused a slight increase in Cd accumulation in the root relative to the control (0.472 mg/kg). From the aspect of phytoextraction, melatonin showed a positive effect in plants pre-treated with this hormone in terms of accumulation of higher amounts of Cd. Increased accumulation occurred in plants treated with both Zn and Cd. Thus, the concentration of Cd in the roots of plants treated with Zn and melatonin was 0.663 mg/kg of the lemon balm root, which is 2 times higher than the plants treated with zinc without melatonin pre-treatment (0.364 mg/kg). A similar effect of increased Cd accumulation was observed in lemon balm leaves, indicating a positive effect of melatonin in the phytoremediation of soil contaminated with heavy metals. The results showed the presence of significantly higher concentrations of Mg and Ca in the leaves relative to the roots (Figure 3C and 3D).

Cd and Zn treatment caused a statistically significant increase in Ca content of the lemon balm root (1.71 and 2.14 mg/g DW compared to the control 1.40 mg/g DW). Melatonin pre-treatment (1.46 mg/g DW), and combination with Cd and Zn ions did not affect the change in Ca concentration relative to control (1.44 and 1.41 mg/g DW, respectively). Compared to controls (15.19 mg/g DW), Ca content in the leaves was significantly decreased in Cd-treated plants (12.73 mg/g DW) and significantly increased in Zn-treated plants, where the concentration of this nutrient increased to 21.85 mg/g (Figure 3C). Exogenous melatonin had a significant inhibitory effect on Ca content in leaves (9.87 mg/g DW). Also, a statistically significant decrease in calcium concentration occurred in the plants pre-treated with melatonin and treated with Cd (8.79 mg/g DW) and Zn (9.16 mg/g DW).

The results show a statistically significant increase in the magnesium content of the Cd-treated lemon balm root (0.15 mg/g DW) and a slight increase in the roots of the Zn-treated plants (0.11 mg/g DW) compared to the control (0.10 mg/g DW) (Figure 3D). Exogenous melatonin pre-treatment did not affect changes in Mg concentration compared to control. There is also no significant change in the content of this macronutrient in the root of melatonin pre-treated lemon balm plants with heavy metal treatment. 

It has been shown that melatonin may be effective in improving the phytoremediative capacity of plants. To investigate the potential links between melatonin pre-treatment and plant tolerance to environmental stress, pea plants were treated with high amounts of copper. The results show that copper contamination had a negative effect on the pea plants growth. However, the addition of melatonin to soil significantly increased their tolerance to copper contamination, and thus increased their survival. Based on published data, the use of melatonin to enhance the phytoremediative capacity of different plants may be a viable and cost-effective approach against environmental contamination [29]. Also, it was proposed and improved the remediation efficiency of poorly contaminated soil with cadmium by treating *Galinsoga parviflora* with 100 µM melatonin. Melatonin enhanced the translocation of Cd from roots into shoots of this plant species. The favorable concentration of melatonin also influenced the increase in biomass. Potato weed is a Cd hyperaccumulator and has a high tolerance to it. Under conditions of low Cd concentration, melatonin not only improved the activity of antioxidant enzymes, but also enhanced the transfer of Cd to the cell wall and vacuoles, removing Cd away from the sensitive parts of the cell and accelerating its absorption [33].

**Figure 4:**
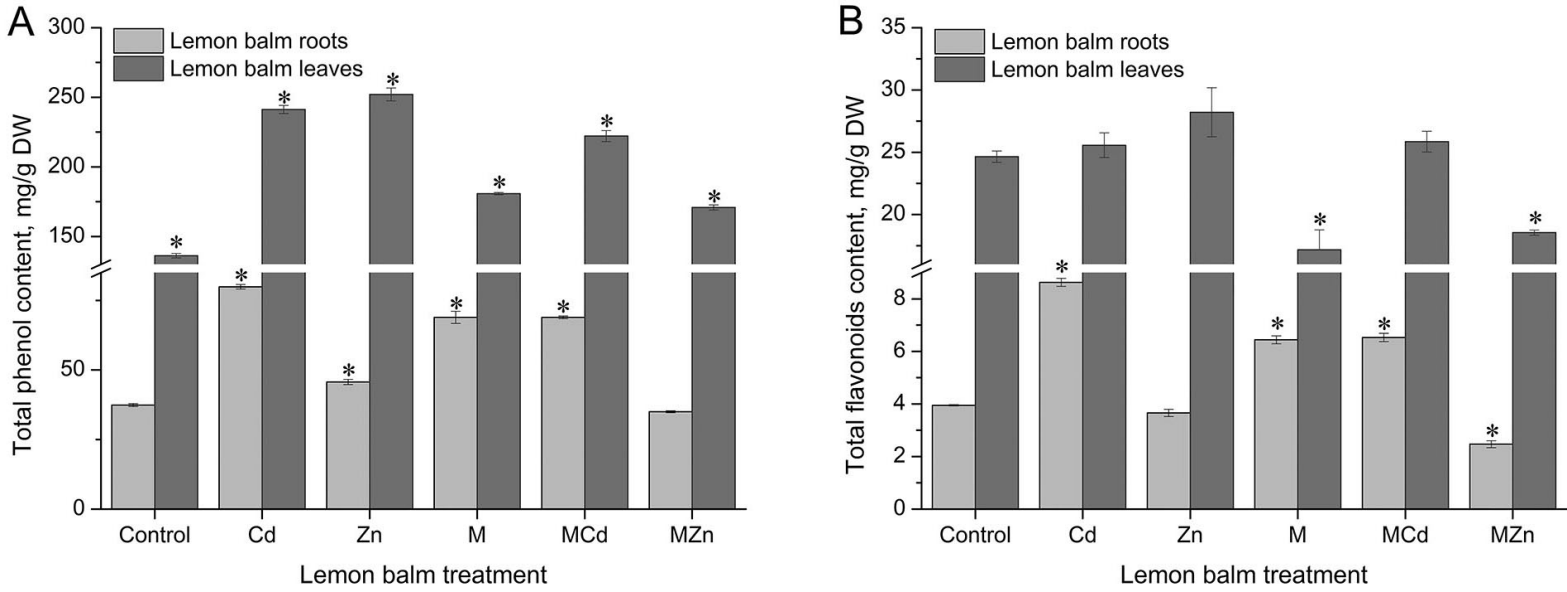
Total phenol (A) and flavonoid (B) content in lemon balm leaves and roots under heavy metal treatment and melatonin pretreatment. The data are presented as means of three replicates. Dots marked with an asterisk differ significantly (Tukey test; p < 0.05). M - melatonin.

The content of phenolic compounds in the lemon balm root ranged from 38 to 80 mg/g of freeze-dried root and 136 to 253 mg/g of freeze-dried lemon balm leaves (Figure 4A). The content of total phenolic compounds in the leaves is three to six times higher than in the roots. According to Tukey test, the differences in the content of total phenols in ethanol extracts of the lemon balm root in all treatments were clearly observed. By the action of Cd and Zn, a statistically significant increase in the content of total phenols was observed relative to untreated plants (79.93 and 45.68 mg/g DW). Melatonin pre-treatment also caused an increase in total phenolics compared to controls (twofold higher content, 68.9 mg/g DW). Melatonin pre-treated plants in Cd and Zn contaminated soil showed statistically significantly higher content of total phenolic compounds compared to control plants, however their content was significantly lower compared to plants treated with heavy metals in which exogenous melatonin was not used (68.89 and 34.99 mg/g DW). Variations in the total phenol content are due to the action of many factors, which in addition to those of genetics; include the area of cultivation as well as many environmental factors [34].

The flavonoid content ranged from 2.5 to 9 mg/g of lyophilized lemon balm roots, and 17 to 29 mg/g of lyophilized leaves (Figure 4B). Lemon balm leaves show three to seven times higher concentration of flavonoid than its root. Cd ions in the lemon balm roots contributed to an increase in flavonoid concentration compared to control, untreated plants, by double the value (8.63 mg/g DW). The same stimulatory effect of Cd occurs in plants pre-treated with exogenous melatonin (6.53 mg/g DW). Zn, on the other hand, had an inhibitory effect on flavonoid content in plants with and without exogenous melatonin addition (2.47 and 3.66 mg/g DW). Treatment of the plants with melatonin alone showed a significant increase in flavonoid content relative to untreated plants (6.44 mg/g DW). The increase in concentration is statistically significant. Completely different results were obtained in leaves. Heavy metal ions contributed to a slight increase in flavonoid concentration (25.56 mg/g DW) compared to untreated plants (24.65 mg/g DW), but the increase was not statistically significant. Treatments with exogenous melatonin, melatonin and Zn caused a significant decrease in flavonoid content (17.17 and 18.55 mg/g DW), whereas no change was observed in melatonin treated plants in Cd-contaminated soil. 

In accordance to our study, the influence of melatonin on the phenolic compounds content in berries has been examined [35]. The total phenols, flavonoids and proanthocyanides were gradually improved with fruit ripening with a maximum of 77 days in the fruit crust. Also, melatonin treatment significantly improved the content of total phenols, flavonoids and proanthocyanides [35]. Also, 15 µM melatonin stimulates the total phenol and flavonoid content of bitter orange seedlings, *Citrus aurantium* L [36]. The results show an increase of almost twofold over control, untreated plants. Melatonin has been shown to have many physiological functions in plants, and the most investigated function is the prevention of oxidative damage caused by various abiotic stressors such as salinity [37], low temperatures [38], and cadmium toxicity [39]. It is suggested that melatonin should be classified as an antioxidant that acts in the mitochondria as it achieves its antioxidant capacity by directly detoxifying reactive oxygen species and reactive nitrogen species and indirectly stimulating antioxidant enzymes, suppressing pro-oxidant enzymes activity [7]. It has been shown that exposure to heavy metals results in changes in protein content, and it is likely that some of these proteins have a catalytic function. Heavy metals also cause changes in the catalytic activity of the enzyme due to the high binding affinity for sulfhydryl or other groups from their active center, leading to a decrease in activity or complete inhibition [40].

**Figure 5:**
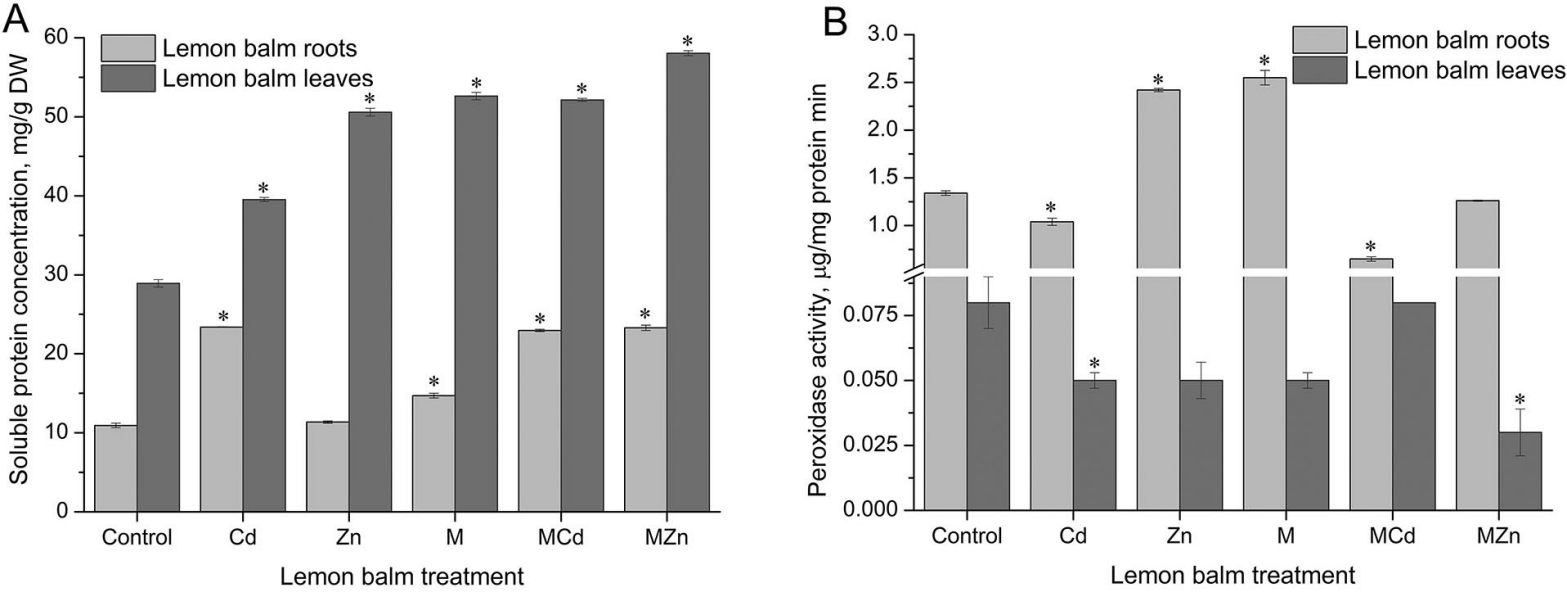
Total soluble proteins content (A) and peroxidase activity (B) in lemon balm leaves and roots under heavy metal treatment and melatonin pre-treatment. The data are presented as means of three replicates. Dots marked with an asterisk differ significantly (Tukey test; p < 0.05). M - melatonin.

Analysis of heavy metal and exogenous melatonin influence on soluble protein content (Figure 5A) shows significant increase in the protein content of the lemon balm root when exposed to cadmium ions (23.38 mg/g DW) compared to the control (10.85 mg/g DW). Melatonin pre-treatment also had similar stimulative effect (14.70 mg/g), as well as melatonin with cadmium ions (22.95 mg/g DW), and zinc ions (23.29 mg/g DW). Lemon balm leaves show up to three times higher concentration of soluble proteins relative to the root. Also, all treatments increased the soluble protein content. The most significant, twofold increase occurred in the leaves of plants pre-treated with exogenous melatonin (53.52 mg/g DW). 

It has been shown that the concentration of endogenous melatonin may be related to protein content in plants. Young mulberry leaves have a higher protein content compared to older leaves. Several amino acids, including tryptophan, which is a precursor of melatonin, are also present in mulberry leaves [41]. Soluble protein concentration increases significantly in wheat leaves, subjected to low temperatures. An increase by 12% was obtained. Application of exogenous melatonin resulted in an even higher (20%) soluble protein content. The protein profile determined by SDS-PAGE (sodium dodecyl sulfate electrophoresis on polyacrylamide gel) indicated improved soluble protein content under low temperature stress. Low temperatures stimulated the content of some partially low molecular weight proteins, between 7 and 46 kD, while administration of exogenous melatonin increased the content of many low and high molecular weight proteins [42].

**Figure 6:**
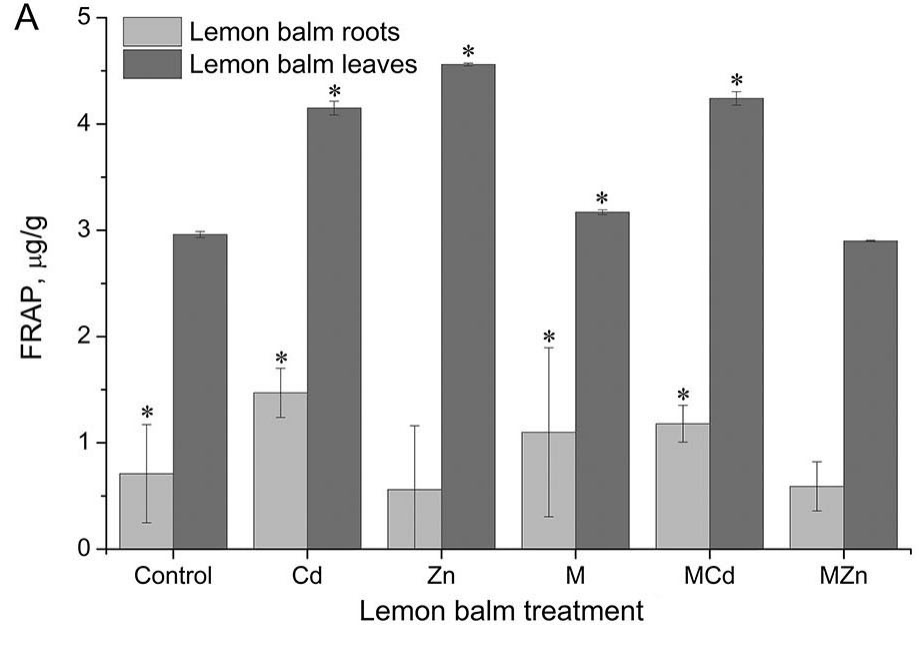

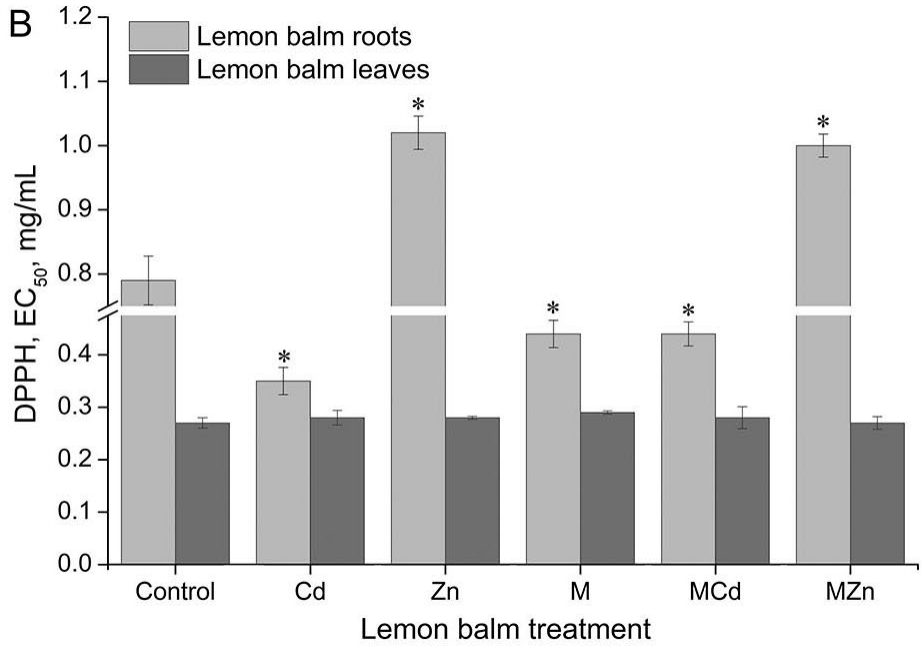

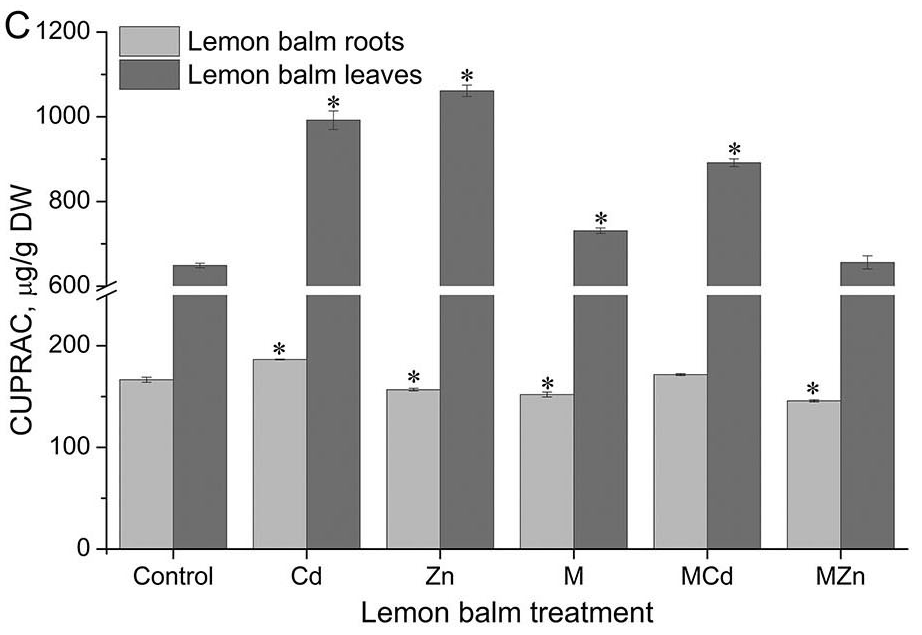
Lemon balm leaves and roots antioxidative activity estimated by FRAP test (A) and DPPH (B) and CUPRAC test (C) under heavy metal treatment and melatonin pre-treatment. The data are presented as means of three replicates. Dots marked with an asterisk differ significantly (Tukey test; p < 0.05). M - melatonin.

In order to overcome the toxicity of heavy metals, plant cells are provided with enzymatic mechanisms by which they eliminate or reduce their harmful effects. Class III peroxidases protect cells against the harmful oxidative action of H_2_O_2_. Monitoring the peroxidase activity in plants under conditions of soil loading with cadmium ions resulted in a decrease in the activity of this enzyme in both, lemon balm roots and leaves. Similar results were obtained earlier in *Valeriana officinalis* L [30]. Under the influence of zinc ions, peroxidase activity increased in the lemon balm root (2.39 µmol mgprotein^-1^ min^-1^), by double the value of untreated plants (1.35 µmol mgprotein^–1^ min^–1^). The lowest peroxidase activity was measured in the roots and leaves pre-treated with melatonin and grown on soil contaminated with cadmium ions (Figure 5B). Although it has been reported that melatonin has an effect on increasing the activity of many antioxidant enzymes under conditions of oxidative stress [43], in our case there was no increase in peroxidase activity. It is possible that the defense activity of peroxidases is based on the protection of plants at the root, where a statistically significant increase in enzyme activity is observed. Also, the applied concentration of cadmium ions could not be as toxic to these highly resistant plant species. A series of biochemical tests were performed to measure the ability of extracts to neutralize free radical species *in vitro* . Effect of heavy metal ions and exogenous melatonin influence on the reducing capacity of lemon balm roots and leaves is given in Figure 6A. 

Exposure of the lemon balm to cadmium ions resulted in an increase in the reducing capacity of its root (1.47 µg/g extract) compared to the control (0.71 µg/g extract) (Figure 6A). An increase was also observed in plants previously treated with exogenous melatonin (1.1 µg/g extract). In contrast, zinc ions caused a decrease in its reducing power (0.56 µg/g extract), which again manifests itself in the roots of plants treated with melatonin with zinc treatment (0.59 µg/g extract). The lemon balm leaves showed three to eight times greater ability to reduce Fe^3+^. In the leaves of plants exposed to ions of both heavy metals, there is a marked increase in reducing ability over control, which can be attributed to the increased defensive effect of plants due to their exposure to stress. Also, pre-treatment with melatonin increased reductive power in the leaves, noting that the increasement was not statistically significant. There was no change in the reduction potency of the melatonin pre-treated leaves grown on soil contaminated with zinc ions (2.89 µg/g extract) relative to the control plants.

The EC_50_ values of the ethanol extracts of the lemon balm leaves varied in the range 0.27–0.29 mg/mL and the roots 0.35–1.02 mg/mL (Figure 6B). The percentage of DPPH radical neutralization in the Cd treated roots is higher than that of untreated roots, indicating that under this stress, a significant increase in the ability to neutralize DPPH radicals is likely due to the activation of antioxidant defense manifested by increased biosynthesis of second metabolites under stress conditions induced by Cd ions. In contrast, the roots grown on zinc-contaminated soil shows a reduced ability to neutralize DPPH radicals. Exogenous melatonin also induced statistically significant increasement in the ability to neutralize DPPH radicals at the root compared to the control. Likewise, the roots of melatonin pre-treated plants show a greater ability to neutralize DPPH radicals with increased Cd ion concentration, but also a reduction in neutralization with increased Zn ion concentration. Significant, two to three times higher ability to neutralize DPPH radicals is observed in the lemon balm leaf. However, no significant increase or decrease in neutralization was observed in any treatment applied compared to untreated plants.

The results of the CUPRAC test (Figure 6C) show good lemon balm ability to reduce Cu^2+^. Cd had stimulatory (186.56 µg/g DW), and Zn inhibitory (156.9 µg/g DW) effect on the Cu^2+^ reduction. Same effect is observed in Zn treated plants pre-treated with melatonin (14.82 µg/g DW). The lemon balm leaves show three to eight times higher ability to reduce Cu^2+^. There is an increased reducing ability in leaves treated with both heavy metals, which can be attributed to the increased defensive effect of the plants due to their exposure to stress. Exogenous melatonin influenced the reduced ability to reduce Cu ions in the lemon balm roots (152.01 µg/g DW) relative to the control (166.54 µg/g DW). Statistically significant increase occurs in the lemon balm leaves (731.00 µg/g DW). Melatonin pre-treatment in combination with heavy metal treatment also shows a significant positive effect, increasing the capacity of both, roots and leaves in this role. 

The results of the study show increased antioxidant activity of plants, especially its leaves in three applied tests. Increased antioxidant activity of the plant leaves can be ascribed to the increased amounts of total phenols, relative to the root. Accordingly, total melatonin values and the content of total phenol compounds were correlated with the value of the reducing capacity of the extracts. A very high degree of correlation between phenol content and the ability to reduce Fe^3+^ ions and the neutralization of DPPH radicals was obtained. When it comes to melatonin, on the other hand, there is no positive correlation. It is possible that the antioxidant activity of melatonin amplifies at the root of the plants analyzed. Because melatonin is highly unstable, it is difficult to monitor its transport in plant organelles and organs [44].

Melatonin acts as a direct antioxidant, and has structural antioxidant capability of reducing many more reactive oxygen equivalents than other natural antioxidants and is not limited by a need for a specific recycling pathway or other metabolites for completion of a redox cycle [45–48]. Recent studies have suggested that exogenous administration of different concentrations of melatonin (0.1, 0.5, and 1 μM) significantly increases the antioxidant capacity of kiwi fruit, *Actinidia chinensis* , exposed to increased salinity (100 mM NaCl). Also, plant damage is significantly reduced. Interestingly, the 0.1 μM melatonin concentration was shown to be most effective [49].

## 4. Conclusion

The protective roles of melatonin to heavy metal induced stress in both lemon balm roots and leaves were investigated by comparing the antioxidative defense system and ROS accumulation levels. Our results demonstrated that the application of exogenous melatonin can alleviate the Cd and Zn damages and improve tolerance in plants through the activation of antioxidative defense systems. Moreover, the signal of exogenous melatonin could be transmitted across organs in the plant. Increased concentrations of endogenous melatonin in plants under different stress conditions imply melatonin involvement in regulating the stress tolerance of plant species [50]. In this highly active molecule lies huge potential for research, understanding basic functions, and opportunities to improve diverse processes in plant cultivation and agriculture.
